# Nutritional therapy in palliative care units: A bibliometric analysis

**DOI:** 10.1097/MD.0000000000041772

**Published:** 2025-03-07

**Authors:** Özlem Öner, Pinar Ayvat, Ali Necati Gökmen

**Affiliations:** a Department of Anesthesiology and Reanimation, Dokuz Eylül University Faculty of Medicine, Subdivision of Critical Care Medicine, İzmir, Turkey; b Department of Anesthesiology and Reanimation , İzmir Democracy University Faculty of Medicine, İzmir, Turkey.

**Keywords:** bibliometric, nutrition, palliative, therapy

## Abstract

This bibliometric review aimed to provide a general picture and systematic mapping of research trends in palliative care (PC) and nutrition internationally. The Web of Science (WoS) database was searched on May 7, 2024, for original articles focusing on PC and nutrition between 1970 and 2023. Relevant publications were searched using the Thompson Reuters Science Citation Index (SCI) search engine with the keywords “palliative care,” “palliative care unit,” “nutrition,” and “palliative care and nutrition.” A total of 918 articles were found in 391 sources with the participation of 4772 authors. An average of 18.32 citations per article was identified. When analyzed by country, the United States of America (USA) had the highest number of publications (n = 210), followed by Germany (n = 71) and France (n = 69). In the list of the most frequently repeated keywords in the field of PC and nutrition, the most commonly used words were “palliative care” and “end.” Other common terms included “nutrition,” “quality of life,” and “hydration.” Additional frequently used words were “cancer,” “symptoms,” and “management..” This study is the first step toward analyzing and mapping research related to PC and nutrition. It shows that the number of publications related to PC and nutrition has steadily increased between 1991 and 2023. United State of America and European countries are the top publishers and receive the most citations. Hot topics in the field include “end,” “nutrition,” “quality of life,” “hydration,” “cancer,” “symptoms,” and “management,” highlighting the complexity of the subject.

## 1. Introduction

Palliative care (PC) units are specialized facilities aimed at improving the quality of life for patients with life-threatening illnesses, their families, and caregivers as they cope with physical, psychological, social, or spiritual challenges.^[1]^ The need for PC has increased globally due to the burden of infectious diseases, as seen during the pandemic, as well as the rise in chronic illnesses and aging populations.^[2]^ Moreover, PC is essential not only for adults but also for individuals of all age groups, including children with severe incurable diseases.^[3]^ In fact, receiving PC services is a human right for those in need of such care.^[4]^ For healthcare professionals, it is an ethical duty to provide PC services to these patients, who may have chronic illnesses or be in the final stages of life, by managing symptoms early, alleviating suffering, and respecting human dignity.^[5]^ Each year, approximately 56.8 million people, including 25.7 million in their final year of life, require PC.^[1]^ Unfortunately, only 14% of patients in need of PC can access these services, indicating that the global demand for PC will continue to rise.^[1]^

PC patients often suffer from severe symptoms that negatively affect their quality of life.^[6]^ Among these, nutritional deficiencies are undoubtedly one of the most critical.^[7]^ Malnutrition can impact daily activities and quality of life, increase complication rates, morbidity, and even mortality; hence, its early detection and treatment are essential.^[8]^ Nutritional support is a crucial aspect of PC for patients with life-threatening illnesses.^[9]^ Proper nutritional support is important not only in meeting the physical needs of the body but also in providing social, cultural, and psychological support to patients.^[10]^ With the growing need for PC units and the significance of nutritional therapy, it raises the question of whether research and publication awareness on this topic has been established worldwide.^[11]^

In bibliometric analysis research, various quantitative methods are used to measure, track, and analyze the published scientific literature over extended periods.^[12]^ This method aims to process large volumes of research published over a long period with limited resource and time investment.^[13]^ Bibliometric analysis measures productivity (e.g. the number of published articles), impact (e.g. citation count, journal impact factor [IF]s), and collaboration between countries, institutions, and authors.^[14]^ It helps in understanding the growth and trends in scientific research in a specific field and contributes to the development of health initiatives.^[15]^ The Web of Science (WoS) online database, which we used for our research, is not freely accessible and contains journal articles from all major disciplines dating back to 1900, providing tools for generating representative figures.^[12]^

The aim of this research is to assess the current state of interest in nutrition, which we consider a significant issue in PC, as the global need for and interest in PC continues to grow. For this purpose, we intend to examine the most cited articles on PC and nutritional therapy in PC units in terms of authors, journals, countries, and recurring keywords (hotspots) using the Thompson Reuters WoS Citation Indexing (SCI) database, and to shed light on the direction of science in this field by determining citation trends.

## 2. Methodology

### 2.1. Search strategy

The Thompson Reuters SCI citation indexing database was searched on May 7, 2024, following approval from the ethics committee (Decision No: 2023/196, Approval Date: December 27, 2023). Informed consent was not required due to the design of the study. We utilized the WoS (Clarivate Analytics, Philadelphia) database because it includes various bibliometric indicators and literature from different disciplines.^[16]^ In this study, the citation rates, authors, journals, countries, international collaborations, and hotspots of publications on “PC and nutrition” were evaluated. The data collection and analysis process is detailed below.

### 2.2. MeSH terms strategy

To determine the most cited articles at the international level, as well as citation trends and hotspots in the field of “PC and nutrition,” appropriate search terms were compiled. Relevant publications were searched using the Thompson Reuters SCI search engine with the keywords “palliative care,” “palliative care unit,” “nutrition,” and “palliative care and nutrition.”

### 2.3. Data collection and analysis

The search was limited to English-language publications, and original research articles were included, while other types of publications were excluded. Performance analysis, collaboration analysis, and scientific mapping were conducted on authors, citation frequency, author collaboration, and keywords to gain insights into this scientific field. The search timeframe was set between January 1, 1970, and April 31, 2023. The initial search, using the selected keywords, yielded 379,595 results. Since our aim was to investigate publications specifically related to PC, we refined the search using “Palliative Care” under Citation Topics Micro and Citation Topics Macro in the search engine, narrowing it down to 918 results. For analysis, the exported data was saved in “full records and cited references” format as plain text, tab-delimited format, and Excel outputs. Analysis was performed using Excel, the Bibliometrix R package (version 4.1.2), and an online bibliometric application (https://bibliometric.com/app) for data tabulation and visualizations.

### 2.4. Performance analysis

In the performance analysis, data on the growth of publications, the most active countries and institutions, the most cited journals, and the mapping of publications and keywords were analyzed to measure the main performance of the research in this field. A citation analysis was conducted at this stage. To determine the 2023 IF of each journal, the Journal Citation Reports dataset was used (https://clarivate.com/products/scientific-and-academic-research/research-analytics-evaluation-and-management-solutions/journal-citation-reports). The Hirsch index (H-index) and IF were employed to demonstrate the impact of the publications. The H-index is a valuable measure for evaluating academic productivity.^[17]^ It can be applied to assess the academic productivity of a department, university, country, scientist, or journal. The IF is a metric that demonstrates the impact of journals in their fields, calculated by examining citations of articles in scientific journals.

### 2.5. Collaboration analysis

To highlight the scientific collaboration between countries where the publications originated, a collaboration analysis was performed. For this analysis, bibliometrics such as self-citation practices (SCP), multiple country publications (MCP), citation frequency (Freq), and the ratio of MCP were used.

### 2.6. Scientific mapping

Scientific mapping is a system that represents relationships between “disciplines, fields, specialties, and individual articles or authors.” In our study, the most cited authors, scientific collaborations, and co-word analysis were conducted using the Wosviewer method. The mappings illustrating these relationships were created using Vosviewer version 1.6.15 (https://www.vosviewer.com/).

## 3. Results

### 3.1. Performance analysis

**Figure 1.**
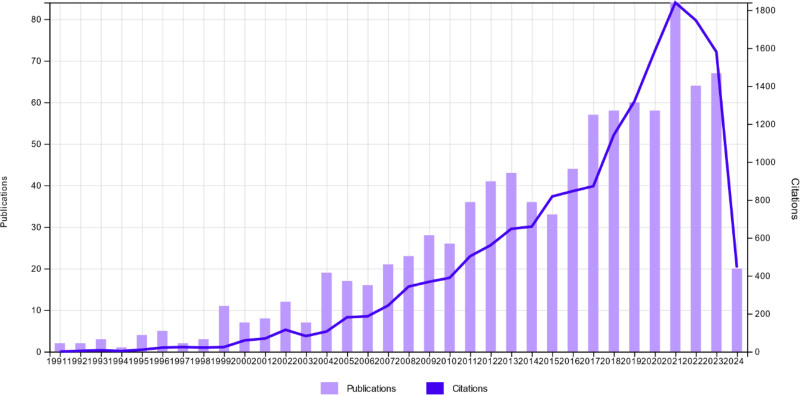
Increasing citation trend over the years.

The Thomson Reuters SCI database was searched for publications written in English between January 1, 1970, and April 31, 2024, on the topics of “Palliative care” and “nutrition.” A total of 918 articles were found in 391 sources with the participation of 4772 authors. An average of 18.32 citations per article was identified. The first publication related to the selected topic was dated 1991. The most cited publication was titled “EFNS guidelines on the Clinical Management of Amyotrophic Lateral Sclerosis (MALS)-revised report of an EFNS task force,” which was cited 746 times and published by Andersen and colleagues in the “European Journal of Neurology” (Table 1). The top ten most cited articles are summarized in Table 1. The annual growth rate of publications on “Palliative care” and “Nutrition” was found to be 7.68% (Fig. 1). The average age of the documents was calculated as 9.04 years. The total number of citations for these articles was 16,815, with an h-index of 58.

**Table 1:** The top 10 most cited articles in the field of palliative care and nutrition.

Yazarlar	Title	Source	Year	Total citations	Assigned cluster	First author institution	Country
Andersen, Peter M.; ve arkadaşlari	EFNS Guidelines on the Clinical Management of Amyotrophic Lateral Sclerosis (MALS) - Revised Report of an EFNS Task Force	European Journal of Neurology	2012	746	Quality-of-life	Umeå University	Sweden
Van Der Steen, Jenny T.; et al	White Paper Defining Optimal Palliative Care in Older People with Dementia: A Delphi Study and Recommendations from the European Association for Palliative Care	Palliative Medicine	2014	583	Palliative care	Leiden University Medical Center	Netherlands
Wang, Ching H.; et al	Consensus Statement for Standard of Care in Spinal Muscular Atrophy	Journal of Child Neurology	2007	572	Quality-of-life	Stanford University School of Medicine	USA
Finkel, Richard S.; et al	Diagnosis and Management of Spinal Muscular Atrophy: Part 2: Pulmonary and Acute Care; Medications, Supplements and Immunizations; Other Organ Systems; and Ethics	Neuromuscular Disorders	2018	338	Quality-of-life	St. Jude Children’s Research Hospital	USA
Sykes, N.; Thorns, A.	The Use of Opioids and Sedatives at the End of Life	Lancet Oncology	2003	255	End	St. Christopher’s Hospice	UK
Carli, Francesco; et al	Effect of Multimodal Prehabilitation vs Postoperative Rehabilitation on 30-Day Postoperative Complications for Frail Patients Undergoing Resection of Colorectal Cancer: A Randomized Clinical Trial	JAMA Surgery	2020	229	Quality-of-life	McGill University Health Centre	Canada
Quill, T.E.; Byock, I.R.	Responding to Intractable Terminal Suffering: The Role of Terminal Sedation and Voluntary Refusal of Food and Fluids	Annals of Internal Medicine	2000	197	End	University of Rochester Medical Center	USA
Allard, Johane P.; et al	Malnutrition at Hospital Admission - Contributors and Effect on Length of Stay: A Prospective Cohort Study from the Canadian Malnutrition Task Force	Journal of Parenteral and Enteral Nutrition	2016	169	Quality-of-life	University of Toronto	Canada
Lundholm, K.; et al	Palliative Nutritional Intervention in Addition to Cyclooxygenase and Erythropoietin Treatment for Patients with Malignant Disease: Effects on Survival, Metabolism, and Function - A Randomized Prospective Study	Cancer	2004	163	Quality-of-life	Sahlgrenska Academy, University of Gothenburg	Sweden
Kelleher, D.K.; et al	Growth and Correlates of Nutritional Status Among Infants with Hypoplastic Left Heart Syndrome (HLHS) After Stage 1 Norwood Procedure	Nutrition	2006	161	Quality-of-life	Children’s Hospital Boston	USA

**Figure 2.**
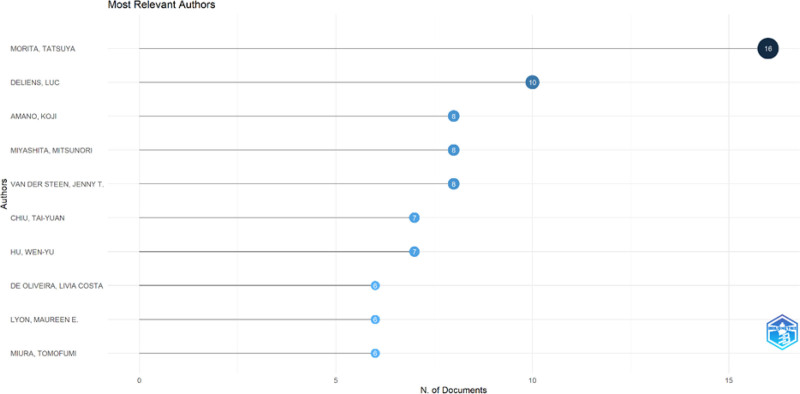
Most relevant authors about PC and nutrition. PC = palliative care.

**Figure 3.**
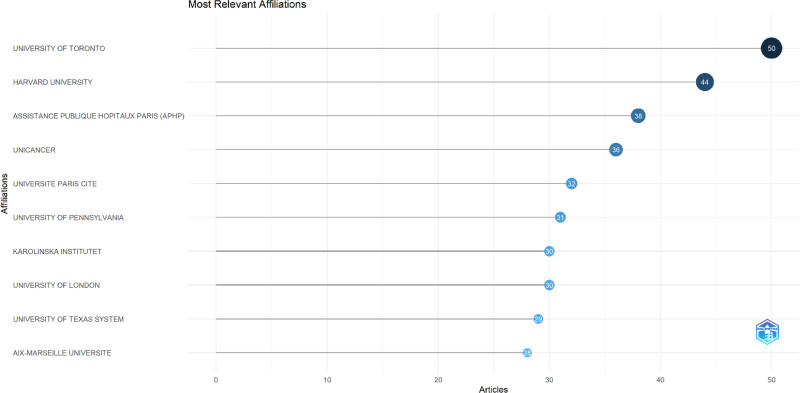
Affiliations about PC and nutrition. PC = palliative care.

**Figure 4.**
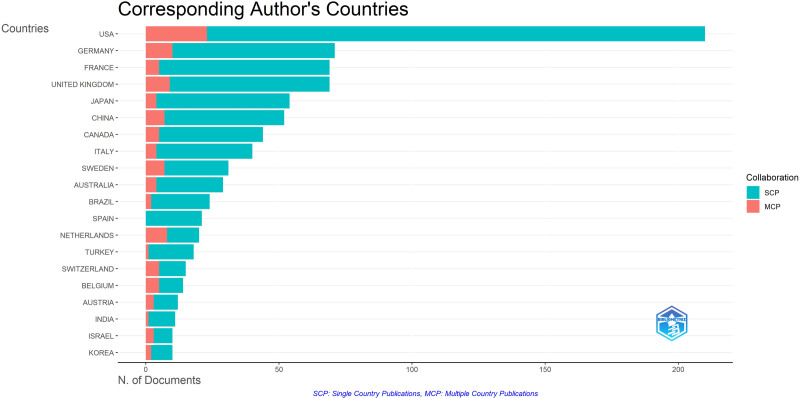
Corresponding author’s countries about PC and nutrition. PC = palliative care.

The top ten authors with the most publications in the field of PC and nutrition are shown in Figure 2. The institutions that published the most articles in the field of PC and nutrition are shown in Figure 3. Furthermore, the proportion of co-authorships in articles involving international collaboration was 13.62% (Fig. 4).

When classified by the number of published articles, citations, and IF, the journal with the highest number of publications was the *Journal of Pain and Symptom Management* (n = 45, average citations = 1307). It was followed by *Supportive Care in Cancer* (n = 37, average citations = 906) and *Clinical Nutrition* (n = 16, average citations = 878; Table 2).

**Table 2:** Top 10 journals with the highest number of citations and articles in the field of palliative care and nutrition.

No	Source	Documents	Citations	Impact factor
1	*Journal of Pain and Symptom Management*	45	1307	3.2
2	*Supportive Care in Cancer*	37	986	3.8
3	*Clinical Nutrition*	16	878	7.7
4	*Palliative Medicine*	26	1196	4.2
5	*American Journal of Hospice & Palliative Medicine*	17	162	1.8
6	*Journal of Palliative Medicine*	27	404	2.4
7	*BMC Palliative Care*	23	239	2.0
8	*International Journal of Palliative Nursing*	8	84	0.9
9	*BMJ Supportive & Palliative Care*	15	72	2.2
10	*Nutrition*	10	376	3.3

When analyzed based on the institutions where the articles were published, the University of Toronto had the highest number of publications (n = 50), followed by Harvard University (n = 44) and Assistance Publique Hopitaux Paris (n = 38; Table 3).

**Table 3:** Collaboration analysis of countries.

Country	Articles	SCP	MCP	Freq	MCP_ratio
USA	210	187	23	0.229	0.11
Germany	71	61	10	0.077	0.141
France	69	64	5	0.075	0.072
United Kingdom	69	60	9	0.075	0.13
Japan	54	50	4	0.059	0.074
China	52	45	7	0.057	0.135
Canada	44	39	5	0.048	0.114
Italy	40	36	4	0.044	0.1
Sweden	31	24	7	0.034	0.226
Australia	29	25	4	0.032	0.138
Brazil	24	22	2	0.026	0.083
Spain	21	21	0	0.023	0
Netherlands	20	12	8	0.022	0.4
Turkey	18	17	1	0.02	0.056
Switzerland	15	10	5	0.016	0.333
Belgium	14	9	5	0.015	0.357
Austria	12	9	3	0.013	0.25
India	11	10	1	0.012	0.091
Israel	10	7	3	0.011	0.3
Korea	10	8	2	0.011	0.2
Mexico	9	7	2	0.01	0.222
Poland	8	8	0	0.009	0
Norway	6	3	3	0.007	0.5
Portugal	6	5	1	0.007	0.167
Denmark	5	4	1	0.005	0.2

Freq = citation frequency, MCP = multiple country publication, SCP = self-citation practices.

When analyzed by country, the United States had the highest number of publications (n = 210), followed by Germany (n = 71) and France (n = 69). When countries were analyzed by total citations and average citations per article, the United States had the highest total citations and average citations per article (TC = 4920, AAC = 23.4), followed by Sweden (TC = 1472, AAC = 47.5) and Canada (TC = 1472, AAC = 47.5). In terms of SCP, the United States had the highest SCP count (n = 187), followed by France (n = 64) and Germany (n = 61). In contrast, regarding MCP ratio, the Netherlands had the highest ratio at 0.4%, followed by Switzerland at 0.333% and Sweden at 0.226% (Table 4).

**Table 4:** Total and average citation numbers of articles published by countries.

Country	TC	Average article citations
USA	4920	23.40
Sweden	1472	47.50
Canada	1334	30.30
United Kingdom	1141	16.50
Netherlands	1132	56.60
Italy	1079	27.00
Japan	785	14.50
Germany	768	10.80
China	761	14.60
France	578	8.40
Switzerland	386	25.70
Australia	354	12.20
Belgium	203	14.50
Korea	202	20.20
Spain	187	8.90
Austria	171	14.20
Czech Republic	140	35.00
Israel	102	10.20
Turkey	96	5.30
Brazil	88	3.70
Denmark	81	16.20
Norway	77	12.80
Chile	76	19.00
South Africa	72	24.00
Singapore	50	10.00

TC = total citations.

### 3.2. Citation growth

Annual citation growth for “Palliative Care” and “Nutrition” is shown in Figure 1. Citation trends indicate steady growth from 1991, the date of the first publication, until 2021, after which there was a decline. The highest number of citations occurred in 2021, with a total of 1800 citations.

### 3.3. Keyword analysis

**Figure 5.**
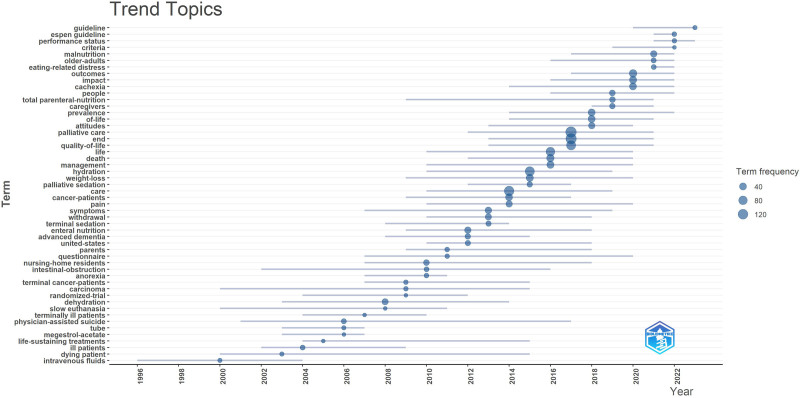
Trend topics about PC and nutrition. PC = palliative care.

**Figure 6.**
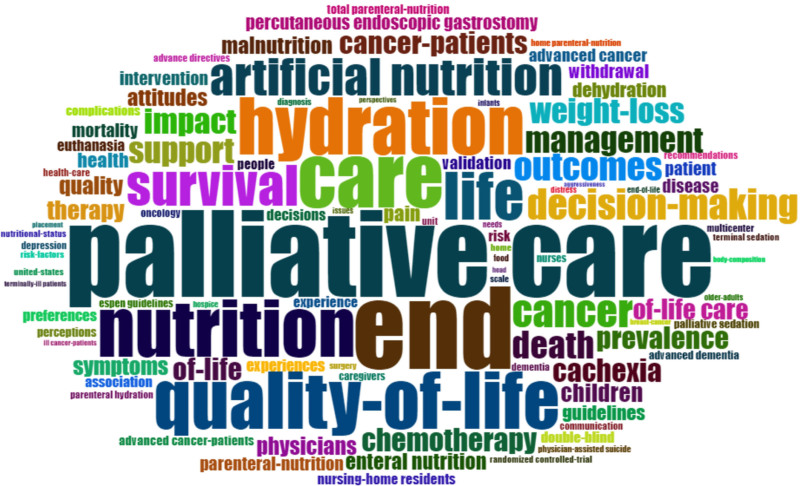
The most repeated words in the article.

In the list of the most frequently repeated keywords in the field of PC and nutrition, the most commonly used words were “palliative care” (157 occurrences) and “end” (149 occurrences). Other common terms included “nutrition” (149 occurrences), “quality of life” (136 occurrences), and “hydration” (114 occurrences; Fig. 5). Additional frequently used words were “cancer,” “symptoms,” and “management.” Figure 6 provides a comprehensive overview of the most common research themes in both PC and nutrition, illustrating the most prevalent topics and their trends over time.

### 3.4. Collaboration analysis

**Figure 7.**
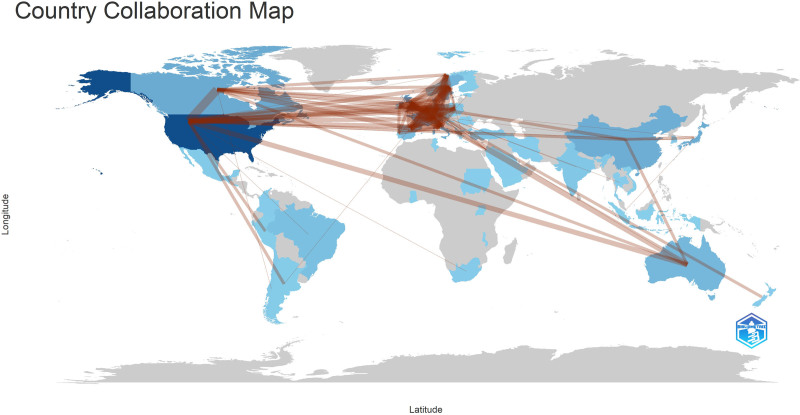
Country collaboration map about PC and nutrition. PC = palliative care.

International collaboration is defined as research contributed by authors from multiple countries. This study presents the frequency of collaborations between countries in Table 2 and Table 5. The countries with international collaborations are visualized using WoSviewer (Fig. 7).

**Table 5:** Collaboration of countries about PC and nutrition.

From	To	Frequency
Argentina	El Salvador	1
Argentina	New Zealand	1
Australia	Netherlands	2
Australia	New Zealand	1
Australia	Poland	1
Australia	Switzerland	1
Australia	Thailand	1
Austria	Estonia	1
Austria	Greece	2
Austria	Ireland	1
Austria	Israel	2
Austria	Norway	1
Austria	Peru	1
Austria	Portugal	1
Belgium	Austria	1
Belgium	Croatia	1
Belgium	Czech Republic	1
Belgium	Denmark	2
Belgium	Finland	1
Belgium	Greece	1
Belgium	Ireland	3
Belgium	Israel	2
Belgium	Norway	2
Belgium	Poland	2
Belgium	Portugal	2
Belgium	Switzerland	5
Brazil	Argentina	1
Brazil	El Salvador	1
Brazil	Peru	1
Brazil	Portugal	1
Canada	Australia	3
Canada	Belgium	2
Canada	Chile	2
Canada	Denmark	3
Canada	Israel	1
Canada	Netherlands	1
Canada	Norway	3
Canada	Poland	1
Canada	Sweden	3
Canada	Switzerland	3
Chile	Colombia	1
China	Australia	3
China	Canada	2
China	Chile	1
China	Korea	2
China	Singapore	1
Czech Republic	Croatia	1
Czech Republic	Slovakia	1
Denmark	Estonia	1
Denmark	Finland	1

PC = palliative care.

### 3.5. Scientific mapping

The scientific map of the most cited authors and the keyword analysis was generated using the Wosviewer method.

## 4. Discussion

This study is a bibliometric review that systematically maps and presents a global overview of research on PC and nutrition from the first publication in 1991 until 2023. Upon conducting a bibliometric analysis of the publications, a total of 918 articles were identified in 391 sources with 4772 authors. These articles, which have been cited 16,815 times in total, have an h-index of 58. Additionally, 13.62% of the authors have demonstrated international collaboration. The citation rate per document is 18.32, and the annual growth rate of 7.68% reflects the high demand for research in PC. The average age of the documents, 9.04 years, suggests that nutrition plays an important role in PC, which is evidenced by 2079 author keywords. An analysis of the most frequently used keywords in PC identified “end,” “nutrition,” “quality of life,” and “hydration” as hot spots.

Measuring the quality of care provided to terminally ill patients is an important indicator that enables healthcare providers and policymakers to monitor and improve the care delivery process. Quality indicators can identify good care and potential problems.^[18]^ Bibliometric review is an appropriate approach to mapping a research field and provides a comprehensive picture of the development and current status of a field over time.^[19]^ In this study, we found that the most cited publication is titled “EFNS guidelines on the Clinical Management of Amyotrophic Lateral Sclerosis (MALS)—revised report of an EFNS task force” published in the *European Journal of Neurology*.^[20]^ This article emphasizes that every effort should be made to preserve the autonomy of patients with Amyotrophic Lateral Sclerosis throughout the course of the disease. It highlights the importance of PC and nutrition for this patient group and suggests early collaboration with patients and their caregivers, with respect for their social and cultural backgrounds and dignity, particularly through advance directives for end-of-life care. The fact that this article has received the most citations may be explained by its reflection of the core spirit of PC. The second most cited study, published by Van der Steen et al, is titled “White paper defining optimal PC in older people with dementia: a Delphi study and recommendations from the European Association for PC” and appeared in *Palliative Medicine*.^[21]^ This article aims to identify the PC needs of patients with dementia and their families, a disease with no curative treatment. The consensus reached includes recommendations on patient-centered care, communication, shared decision-making, symptom management, care goal setting and advance planning, continuity of care, psychosocial and spiritual support, family involvement, healthcare team training, timely recognition of death prognosis, avoiding overly aggressive, burdensome, or futile treatments in nutrition and dehydration, and the feasibility of PC. The third most cited publication is titled “Consensus Statement for Standard of Care in Spinal Muscular Atrophy” by Wang et al, which sheds light on the critical issue of PC and nutrition in children with spinal muscular atrophy who may be dependent on their families for care.^[22]^

Our bibliometric analysis revealed a significant increase in research on PC, particularly from the first publication in 1991 until 2021. The upward trend in publications supports previous research. There are several possible explanations for this result. It could be related to the aging world population and the growing demand for PC, or the peak in publications during the pandemic period, suggesting a connection to the increased burden of infectious diseases.^[23,24]^ On the other hand, it may be related to countries responding to the WHO’s calls for PC policies and the official recognition of PC as a medical specialty in several countries.^[25]^

When articles were evaluated by country, the USA was the global leader in PC research with 210 articles and 4920 total citations. It was followed by Germany, France, and the United Kingdom. Sweden, with a lower number of articles, showed a notable collaboration effort with an MCP ratio of 0.226% and 1472 total citations. Similarly, the Netherlands and Switzerland stood out with high MCP ratios of 0.4% and 0.333%, respectively, indicating strong international research collaborations. In contrast, Poland and Spain showed lower MCP ratios, indicating that single-country publications were more common. The high number of articles from the USA and Europe can be attributed to the fact that many of the authors contributing to PC research work at institutions in these regions, and the study was limited to articles published in English.^[26]^ The high productivity of PC-related publications in these countries is consistent with previous research.^[14]^ Additionally, institutionally, the first PC unit was established in the United Kingdom about 50 years ago.^[27]^ Similarly, the USA has a long history of PC development, with two-thirds of US hospitals offering PC services by 2010.^[28]^ However, Brazil and Turkey showed significantly lower total citations and average citations, indicating that research from these countries had relatively lower impact. Another study found that despite an increase in the number of articles published in some countries, the citation rates remained low. For example, in India, the increase in publication volume was attributed to regulations requiring graduate students to publish in indexed journals, leading to low citation rates.^[29]^ This requirement in India aims to promote institutional PC development.^[30]^ It seems that countries’ institutional PC policies parallel academic indicators such as publication numbers and citation rates. According to the Turkish Ministry of Health, a total of 90,648 palliative patients were served in 437 PC Centers with a total of 6397 beds in the first 6 months of 2023.^[31]^ While this number is significant, considering the country’s population, we believe that Turkey still has a long way to go in the field of PC.

In terms of the specific frequency of terms related to PC and nutrition and their changes over the years, between 1991 and 2023, keywords such as “palliative care,” “end,” “hydration,” and “nutrition” were found to be increasingly repeated. Additionally, “cancer,” “symptoms,” and “management” were identified as hot spots. The significant increase in hydration and fluid management publications until 2015 and their continuation until 2019 is evidence of the topic’s importance. Similarly, the notable interest in “weight loss” from 2009 to 2020 suggests that the difficulty of managing nutrition in PC has been widely acknowledged by authors. In relation to PC, sedation, total parenteral nutrition, and cachexia became recognized in the early 2010s and remained part of core research topics until 2022. Lastly, “quality of life,” “care,” and “pain” were common themes throughout all years, aligning with the findings of other studies on PC. Although PC is a comprehensive approach focused on all age groups and diseases with life-limiting conditions, most PC-related research has been found to focus on adult cancers and nutritional issues.^[8]^ This could be due to the fact that adult cancer diseases constitute the largest group of life-limiting diseases, and their outcomes are dramatic.^[32]^ More than one-third of hospitalized cancer patients either die or are transferred to nursing homes.^[33]^ A survey reported that unplanned hospitalizations for patients with advanced cancer strongly predicted a median survival of <6 months.^[34]^ Therefore, it is not surprising that PC publications are cancer-focused, as in our study. These findings are consistent with a systematic review evaluating international PC research priorities in 2020.^[35]^ Additionally, the global dominance of cancer can explain why oncology continues to be the driving force behind the development of new PC services in many countries.^[36]^ Nevertheless, determining patient needs and improving quality of life and comfort is the primary goal of PC, which is much more important than making accurate prognostic predictions of a patient’s survival.^[37]^ Most patients with life-limiting illnesses develop potentially debilitating symptoms during the course of their illness.^[38]^ Based on the hot spots and trending topics in this study, it was determined that PC and nutrition research has focused on the management of specific symptoms. Symptoms such as constipation, cough, shortness of breath, dry mouth, nausea, vomiting, and fever have received little attention, consistent with previous research.^[11]^ On the other hand, although survival and mortality were not sufficiently emphasized in previous PC studies, the word “end” was one of the most frequently repeated terms in this study. This may be related to the difficulty of providing PC nutrition support to cancer patients with advanced, progressive, and incurable conditions.^[39]^

### 4.1. Limitations and strengths

This bibliometric analysis is limited to original articles available in the Web of Science (WOS) database. Additionally, only articles written in English were included in the analysis, which may somewhat limit the global data analysis. Evaluating the strength of an article based solely on its citation count may prevent a more holistic evaluation.

On the other hand, the analysis of the publications by country based on multiple bibliometric parameters is a strength of this study.

## 5. Conclusion

This study is the first step toward analyzing and mapping research related to PC and nutrition. It shows that the number of publications related to PC and nutrition has steadily increased between 1991 and 2023. The USA and European countries are the top publishers and receive the most citations. Institutionally, countries that provide effective PC services continue to lead academically. Hot topics in the field include “end,” “nutrition,” “quality of life,” “hydration,” “cancer,” “symptoms,” and “management,” highlighting the complexity of the subject.

## Author contributions

**Conceptualization:** Özlem Öner.

**Data curation:** Özlem Öner, Pinar Ayvat.

**Formal analysis:** Özlem Öner.

**Investigation:** Özlem Öner, Pinar Ayvat.

**Methodology:** Özlem Öner, Pinar Ayvat.

**Supervision:** Özlem Öner, Ali Necati Gökmen.

**Validation:** Özlem Öner, Ali Necati Gökmen.

**Visualization:** Özlem Öner, Ali Necati Gökmen.

**Writing – original draft:** Özlem Öner, Ali Necati Gökmen.

**Writing – review & editing:** Özlem Öner.
